# The Chemical Imitation Game: Navigating Spaces of Meaning in Language and Chemistry

**DOI:** 10.1021/acs.jcim.6c01773

**Published:** 2026-08-26

**Authors:** Stefano Moro

**Affiliations:** Molecular Modeling Section (MMS), Department of Pharmaceutical and Pharmacological Sciences, University of Padova, Padova 35131, Italy

## Abstract

The historical evolution of chemistry bears striking parallels to the evolution of language. Both disciplines began as domains governed by tacit practitioner knowledge, underwent successive stages of formalization through systematic symbolic representations, and have ultimately converged toward geometric frameworks that enable machine-guided exploration. From Lavoisier’s nomenclature and Kekulé’s structural formulas to SMILES notation, molecular fingerprints, and learned embeddings, chemistry has progressively transformed molecular representations into navigable spaces. A comparable trajectory can be traced in linguistics, from Dante’s formalization of the vernacular and de Saussure’s structural linguistics to distributed semantic embeddings, large language models, and generative AI. In this Perspective, we argue that these parallels are not merely historical or metaphorical but reflect a deeper representational transition shared by both disciplines: symbols become representations, representations become geometries, and geometries become spaces that can be explored by humans and machines alike. Building on concepts from computational linguistics, molecular machine learning, Free Energy Perturbation (FEP), and diffusion-based generative modeling, we propose a conceptual framework in which molecular properties may be interpreted as forms of chemical meaning emerging from molecular structure. Within this framework, semantic interpolation in latent spaces and alchemical transformations in molecular simulation appear as related strategies for navigating continuous manifolds that connect discrete chemical states. We suggest that modern molecular AI is best understood not simply as a collection of predictive or generative algorithms, but as a new set of tools for exploring chemical meaning within learned representations of chemical space. Recent examples from molecular generative modeling, including our own work on normalizing-flow architectures, illustrate both the promise and the inherent challenges of machine-guided navigation of chemical space. We further propose the concept of a Chemical Imitation Game as a possible framework for evaluating progress in molecular AI beyond syntactic validity and toward chemically meaningful reasoning. This Perspective highlights the opportunities and the limitations of such approaches and argues for a future in which human intuition and machine navigation operate as complementary and mutually reinforcing components of chemical innovation.

## Graphical abstract

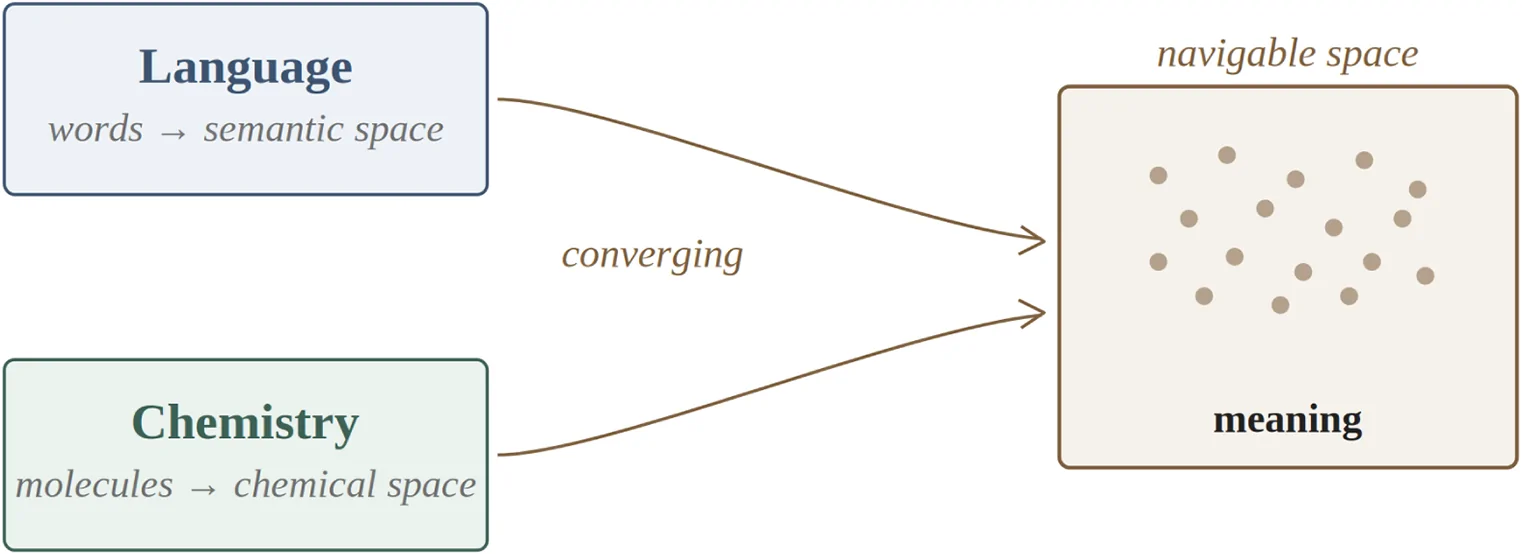

## 1 Introduction—Two Languages, One Question

In 1950, Alan Turing introduced what would become one of the most consequential thought experiments in the history of science—the Imitation Game—asking not whether machines can think, but whether they can produce behavior indistinguishable from that of a human interlocutor.^1^ This shift from essence to representation profoundly shaped the development of artificial intelligence and established a template for evaluating machine capability that has proven extraordinarily fruitful: the question is not what a machine is, but what it can do. Today, a remarkably similar question is emerging in chemistry. As molecular foundation models, generative algorithms, and diffusion-based architectures become increasingly capable of proposing novel molecular structures, we are led to ask not whether machines understand chemistry, but whether their navigation of chemical space reflects forms of reasoning that are functionally analogous to human chemical intuition—and, if so, what that analogy reveals about the nature of chemical knowledge itself.

We propose that this question can be sharpened into what might be called a Chemical Imitation Game: given a set of molecular structures—some generated by a state-of-the-art AI model, others proposed by an experienced medicinal chemist pursuing the same design objective—can an independent expert evaluator distinguish the two sets on the basis of their chemical pertinence, synthetic accessibility, structural originality, and biological plausibility? The Chemical Imitation Game is deliberately more demanding than simple validity checks. Generating chemically valid SMILES strings has become largely a solved technical problem;^2–5^ generating structures that a skilled chemist would recognize as insightful, nonobvious, and synthetically tractable is not. The test is not syntactic but semantic: it probes not whether the machine can speak the language of chemistry, but whether it has anything meaningful to say.

Crucially, the Chemical Imitation Game has not yet been fully passed—though the nature of the remaining gap has been widely misunderstood. Early generative models frequently produced structures with poor synthetic accessibility or unrealistic substructures, but more recent approaches have substantially narrowed this gap: models coupled with retrosynthetic analysis, synthetic-accessibility scoring, and multiparameter property optimization now routinely generate molecules that are both synthetically tractable and pharmacologically reasonable.^6–8^ The frontier, therefore, is subtler than a failure to produce valid or synthesizable molecules. It lies in the simultaneous integration of the many implicit and often tacit constraints—mechanistic plausibility, target-specific selectivity, intellectual-property novelty, and strategic fit within a broader discovery program—that experienced chemists weigh together, and which no single objective function yet captures in full. The game remains open in this specific and important sense. This is not a pessimistic observation: it is a precise diagnosis of where the frontier of molecular AI currently stands, and it points directly toward the research agenda that the remainder of this Perspective attempts to articulate. Just as Turing’s Imitation Game reframed the evaluation of machine behavior in operational terms, the Chemical Imitation Game reframes the evaluation of molecular AI: the relevant measure of progress is not syntactic validity but chemical meaning. We stress that this is a claim about evaluation criteria, not about machine cognition. We do not suggest that generative models possess chemical understanding or intent; the analogy concerns the behavioral indistinguishability of their outputs from those of expert chemists, and we are mindful of the well-documented risks of overattributing human-like capacities to such systems.^9,10^

Language and chemistry are often viewed as unrelated enterprises, yet both are systems for navigating vast spaces of possibility. A word occupies a position in semantic space; a molecule occupies a position in chemical space.^11^ Small symbolic changes can produce large functional ones: cat to bat yields a valid word of entirely different meaning, and CCO to CCN turns ethanol into ethylamine—a valid molecule with markedly different properties. In both domains, the relationship between symbolic proximity and functional proximity is highly nonlinear.

Throughout, we use the term chemical meaning to denote the ensemble of structural, physicochemical, and biological properties that emerge from molecular architecture and determine how a molecule behaves in context. Just as semantic meaning emerges from the arrangement of linguistic symbols, chemical meaning emerges from the arrangement of atoms and bonds—a shared representational challenge across the two domains.

Both disciplines began with the intuitive mastery of practitioners who navigated their spaces without being able to describe how, then underwent successive stages of formalization, and have now reached a point where machines trained on those formal representations generate novel objects—new sentences, new molecules—that inhabit the same spaces as the originals.

Understanding this parallel illuminates what machine-learning models of chemical space actually learn, what they cannot, and what role human intuition must continue to play. The history of chemistry and of language can be read as parallel journeys from symbols to representations, from representations to geometry, and from geometry to navigation. The two arcs and their convergence are shown in Figure 1.

**Figure 1:**
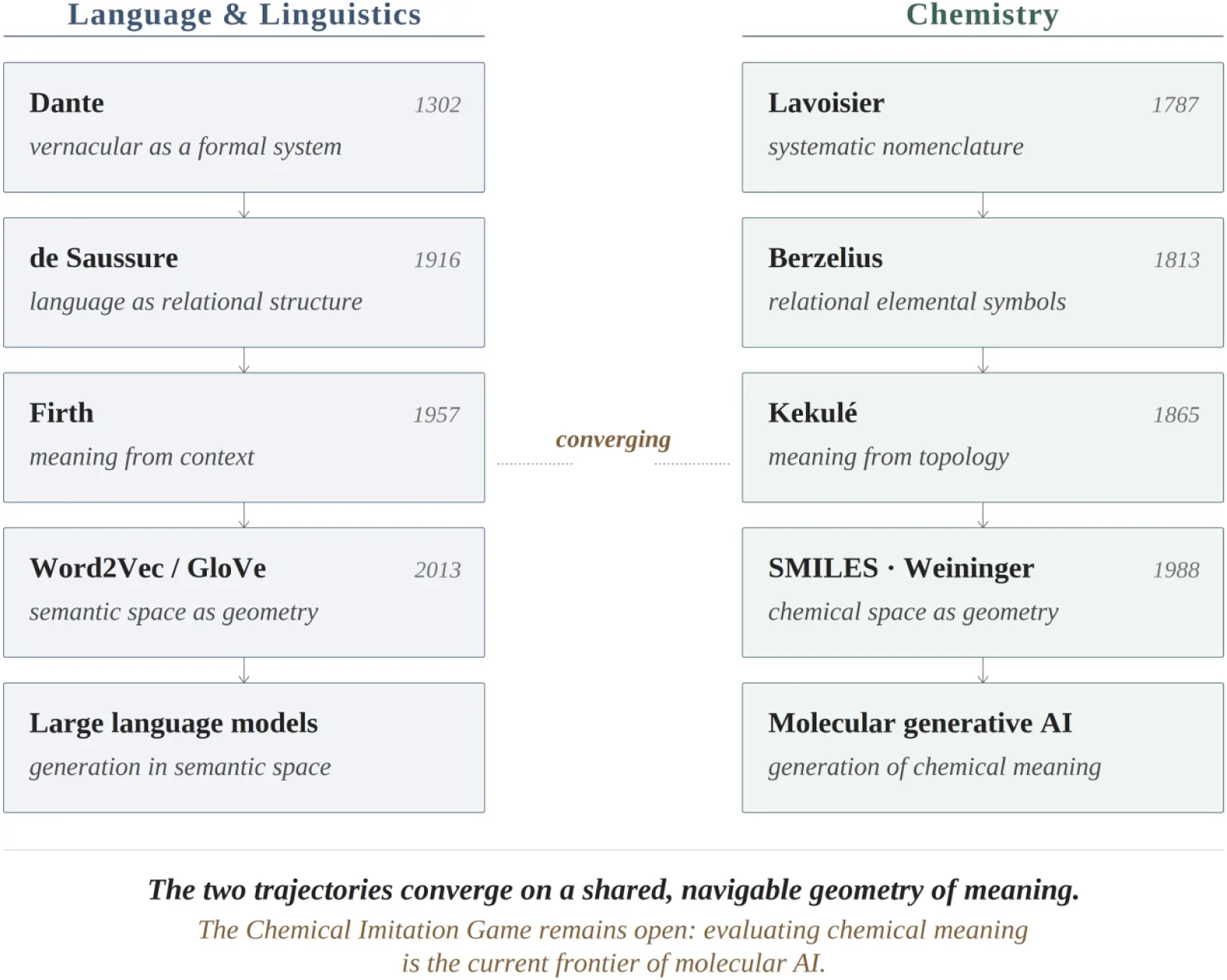
Parallel historical trajectories of linguistics and chemistry, converging toward a shared space of chemical meaning navigated jointly by human intuition and machine intelligence. This figure also serves as the Table of Contents graphic. We note that formalization is not a neutral description of each domain but may itself shape its evolution—plausibly reducing divergence in language while expanding the accessible repertoire in chemistry.

## 2 The Word and the Molecule—Formalizing the Ineffable

### 2.1 Dante and Lavoisier: The Birth of the System

Every mature discipline eventually reaches a point at which its practitioners must agree on a shared vocabulary that encodes the accumulated understanding of the field. In both linguistics and chemistry, this foundational act of formalization was accomplished by figures who stood at the intersection of practitioner mastery and systematic reflection, and whose contributions reshaped the intellectual landscape of their disciplines for centuries to come.

In the early 14th century, Dante’s De vulgari eloquentia offered a formal theory of the vernacular—an attempt to systematize what had been transmitted only through practice. Its central insight was that the vernacular possessed a latent structure that could be analyzed and elevated to a literary standard.^12^ Language, for Dante, was a system waiting to be described.

Four and a half centuries later, Lavoisier’s Méthode de nomenclature chimique (1787) replaced the cacophony of alchemical names with a systematic vocabulary grounded in composition.^13^ For the first time, the name of a substance encoded information about its nature. Chemistry, like the vernacular, was revealed to be a system waiting to be described.

Both Dante and Lavoisier were not primarily discoverers but formalizers: they transformed tacit practitioner knowledge into explicit, transferable representation—the necessary first step toward any computational treatment of their domains.

### 2.2 De Saussure and Berzelius: The Relational Turn

The next conceptual step is best read as an interpretive analogy rather than a strict equivalence. Ferdinand de Saussure proposed that language is a system of differences without positive terms: a word signifies not through any intrinsic property but through its position in a network of relations with all other words.^14^ The unit of meaning is not the sign but the system.

Berzelius’s letter-based notation can be viewed as an analogous insight in chemistry—C, H, O, N.^15^ Far more than shorthand, it defined each substance by its position in a network of elemental compositions: H2O is not merely a name for water but a statement of how water relates to hydrogen, oxygen, and every compound sharing those elements.

In both cases meaning is relational rather than intrinsic—and once meaning is understood as a pattern of relations, it becomes encodable and computable. Chemical meaning, in this sense, is a property of a system rather than of isolated components.

### 2.3 Firth and Kekulé: Context and Topology

Kekulé’s work shifted attention from what a molecule is made of to how its atoms are spatially constituted. This required a shift of attention from the system in the abstract to the concrete patterns in which elements appear—from structure to context, and from composition to topology.

J. R. Firth gave the relational insight its most computationally fertile form: “You shall know a word by the company it keeps.”^16^ This distributional hypothesis—that meaning can be inferred from co-occurrence patterns—is the theoretical foundation of every modern language model.

Kekulé’s 1865 recognition that benzene possessed a cyclic structure was a topological turn of equivalent depth.^17^ Before Kekulé, formulas described composition but not arrangement; the structural formula made explicit that the same atoms, differently connected, constitute entirely different substances. A molecule, like a sentence, is not a list of components but a structure defined by connectivity.

The distributional hypothesis in linguistics and the structural hypothesis in chemistry are, at bottom, the same insight: meaning emerges from pattern and arrangement, not from substance alone. In linguistics this led to distributional semantics and embedding models;^12^ in chemistry, to graph-based representations, fingerprints, and learned embeddings.^13^ In both, structure becomes geometry—the threshold at which each domain becomes not only describable but navigable.

## 3 From Symbols to Geometry—The Birth of Navigable Spaces

The formalizations described in the preceding section created symbolic languages powerful enough to describe their respective domains with precision. But symbolic description, however precise, does not by itself create a space that can be navigated algorithmically. Navigation requires geometry: a representation in which meaningful similarity corresponds to measurable proximity, in which directions in the space carry interpretable meaning, and in which the traversal of a path from one point to another corresponds to a meaningful transformation of the object represented. The conversion of symbolic representations into geometric ones is the decisive step in both the history of computational linguistics and the history of computational chemistry.

In computational linguistics, this transformation was achieved through the development of distributed word representations. The theoretical groundwork was laid by Harris’s distributional semantics,^18^ which proposed that the meaning of a word could be operationalized as a function of its distributional context. The practical realization came with the development of vector space models: representations in which each word in a vocabulary is assigned a vector in a high-dimensional numerical space, with the vectors positioned such that words with similar distributional patterns are geometrically proximate. Early vector space models used term-document or term–term co-occurrence matrices; later techniques, culminating in the Word2Vec and GloVe models developed in the early 2010s, learned dense low-dimensional representations that captured semantic regularities with remarkable fidelity.^19,20^

The semantic space that emerged possessed remarkable properties: analogical relations became vector differences, and semantic similarity became measurable as geometric distance. The meaning of a word became its position in a geometry.

In cheminformatics, the analogous transformation was initiated by the introduction of SMILES notation by David Weininger in 1988.^21^ SMILES—Simplified Molecular Input Line Entry System—linearized the two-dimensional topology of a molecular graph into a one-dimensional character string, encoding atoms as element symbols and bonds as connection rules according to a compact and unambiguous grammar. This linearization was consequential: it made molecular structures parseable by the same computational tools that had been developed for text, enabling the application of string-processing, pattern-matching, and eventually machine learning methods to molecular data. From SMILES strings, it became possible to compute molecular fingerprints—high-dimensional binary vectors encoding the presence or absence of structural features—and to embed molecules in a chemical space in which structural and pharmacological similarity corresponded to geometric proximity.^22^

The Morgan algorithm extended-connectivity fingerprints (ECFPs), developed by Rogers and Hahn, provided a particularly powerful representation of molecular topology: each bit in the fingerprint encodes the local chemical environment of an atom up to a given radius, so that molecules with similar local structural patterns are represented by similar fingerprint vectors.^22^ The Tanimoto coefficient between two fingerprint vectors provides a quantitative measure of molecular similarity that correlates well with biological activity similarity across diverse chemical series—a molecular analogue of the cosine similarity measure used in semantic space. Chemical space, like semantic space, had become a geometry in which similarity was measurable and navigation was in principle possible.

The birth of these navigable spaces—semantic and chemical—marks a decisive transition in the history of both disciplines. Before this transition, the objects of each domain could be described and compared, but comparison required human judgment and was not scalable. After it, comparison became algorithmic: given two molecules or two words, a computer could in principle assess their similarity, cluster them with related objects, interpolate between them, and eventually generate new objects that occupied desired positions in the learned space. Once a domain has been formalized, the question is no longer whether machines can learn to navigate it, but only how well.

The linguistic analogy extends in an unexpected direction. Just as water, eau, acqua, and point to the same reality through different symbols, the formal languages of chemistry—SMILES, InChI, SELFIES, molecular graphs, 3D representations—encode the same chemical meaning through different grammars. A molecule is no more identical to its SMILES string than water is to the sound of its name.

Empirically, molecular representation-learning studies suggest that models trained on different chemical representations can organize molecules in broadly consistent ways, with related structural and functional classes occupying comparable regions of the learned space.^23^ Whether these observations extend to a genuinely universal molecular embedding space—a single shared geometry into which multiple chemical languages map without loss—remains, we emphasize, a speculative and open question rather than an established result. We raise it here not as a demonstrated fact but as a hypothesis that, if confirmed, would support the broader thesis of this Perspective: that chemical meaning is more fundamental than any particular symbolic system used to express it.

### 3.1 Two Vocabularies, Two Growth Curves

The parallel becomes even more striking quantitatively. How has the vocabulary of language grown over time, compared to the molecular vocabulary of chemistry? The contrast reveals why machine navigation of chemical space has become not merely useful but increasingly necessary.

Chaucer’s working vocabulary was roughly 8,000 words; Shakespeare’s about 20,000. The first Oxford English Dictionary (1928) cataloged over 400,000 lemmas, and analyses of large digitized corpora estimate the total English vocabulary at around one million words, growing by a few thousand per year.^24^ Language grows steadily and organically.

The chemical vocabulary follows a different trajectory. The CAS Registry required 40 years to reach its first 25 million substances (2005), then added the next 25 million in only four. By 2015 it held 100 million; today it exceeds 290 million, growing by roughly 15,000 new substances per day (Figure 2).^25^

**Figure 2:**
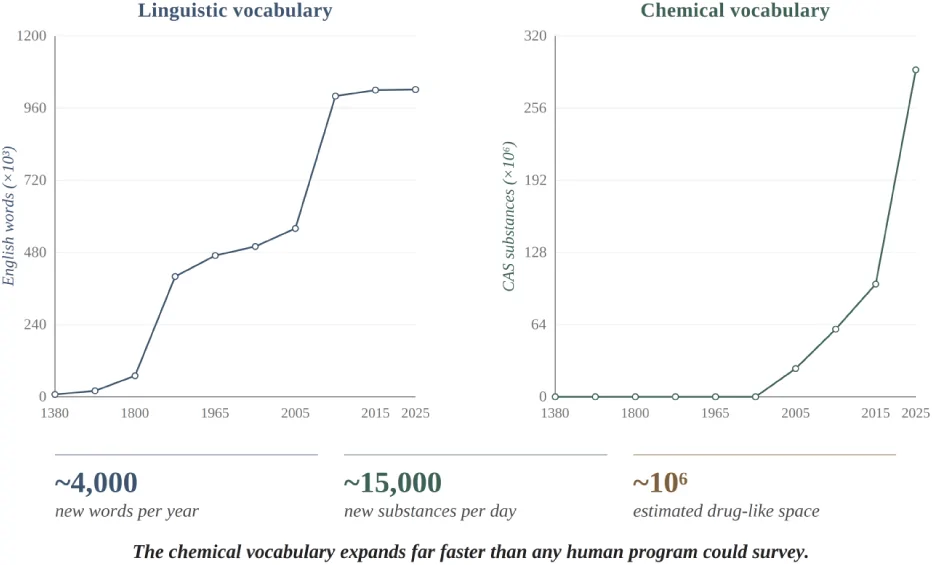
Growth of the linguistic vocabulary (English words, left) and the chemical vocabulary (CAS Registry substances, right) from the 14th century to the present. The chemical vocabulary grows exponentially and far faster than any human program could survey.

The contrast is extraordinary: the linguistic vocabulary grows by thousands of words per year, the chemical vocabulary by millions of structures. And the space itself is vastly larger—the number of synthetically accessible drug-like molecules is estimated at around 10^60^.

This asymmetry has a profound implication. In language, machines navigate a space humans have largely mapped through millennia of use. In chemistry, they must navigate a space growing faster than any human program could survey, and still almost entirely unexplored. Here machine navigation is not merely an augmentation of human effort—it makes exploration of a qualitatively different scale possible.

### 3.2 From Typographical Errors to Molecular Mutations

Consider cat to bat: a single-character substitution preserving validity while producing a large semantic displacement—the two words share almost no neighbors, and their embedding cosine distance far exceeds that between cat and kitten.

The chemical analogue is CCO (ethanol) to CCN (ethylamine): a single-atom substitution preserving validity while shifting hydrogen-bonding, p*K*_a_, permeability, and biological behavior. The Tanimoto similarity between their fingerprints is lower than between ethanol and propanol, despite the smaller string edit.^26^ Symbolic proximity does not predict functional proximity (Figure 3).

**Figure 3:**
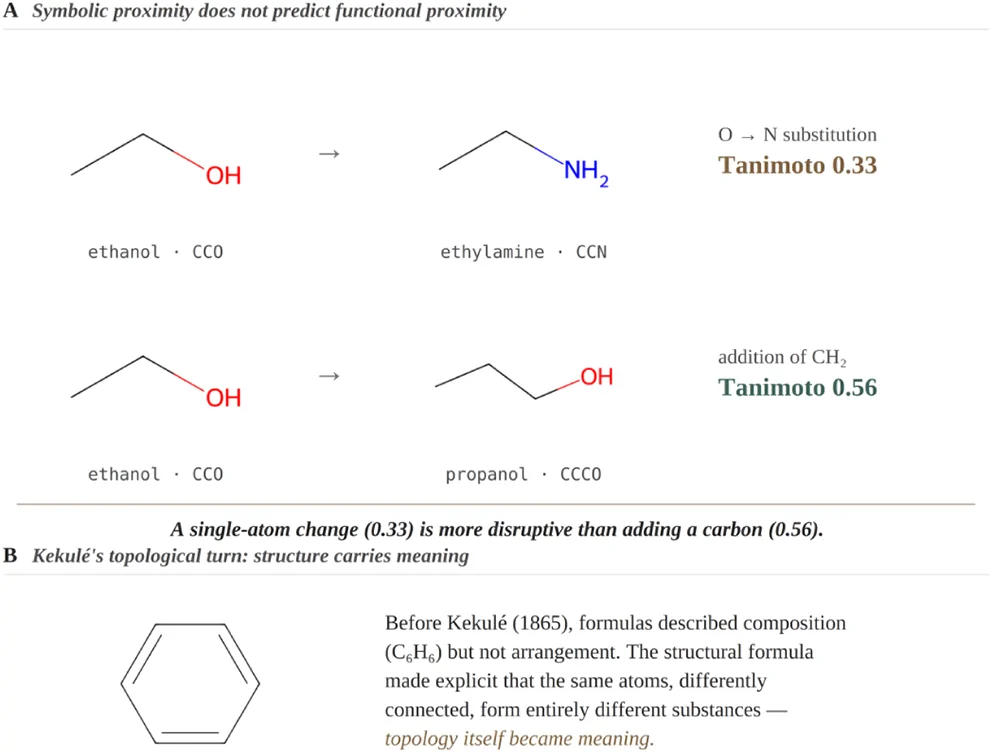
Chemical structures illustrating that symbolic proximity does not predict functional proximity. (A) A single-atom substitution (ethanol → ethylamine, O → N) yields a lower Morgan-fingerprint Tanimoto similarity (0.33) than adding a carbon (ethanol → propanol, 0.56), despite the smaller symbolic change. (B) Kekulé’s structural formula for benzene and its SMILES encoding (c1ccccc^1^): topology itself carries chemical meaning.

This has direct implications for molecular AI. A model that walks randomly in SMILES-string space implicitly assumes symbolic distance tracks functional distance—precisely what the cat-to-bat case refutes. The most effective generative models therefore navigate in learned latent spaces trained to preserve functional relationships, rather than in raw string space.

Distinguishing conservative from transformative substitutions—in both linguistic and chemical space—is exactly the tacit knowledge experienced chemists accumulate through practice, and precisely what learned geometries must recover to be useful. This is the difference between navigating chemical space and understanding it.

## 4 Machines That Generate—LLMs and Molecular AI

The development of deep generative models over the past decade represents the culmination of the trajectories traced above. In natural language processing, the transformer architecture introduced by Vaswani and colleagues in 2017 enabled the training of large language models on unprecedented scales of text data, with qualitatively new emergent capabilities.^27^ These models learn not only the statistical regularities of word co-occurrence—the distributional signal that word embeddings had already captured—but also the long-range dependencies, hierarchical structure, and contextual disambiguation that characterize natural language at the level of sentences, paragraphs, and documents. The result was a generation of models that could produce fluent, contextually appropriate, and sometimes genuinely novel text across a wide range of domains, genres, and tasks—not by retrieving stored text but by sampling from a learned probability distribution over sequences of tokens.

The significance of these models lies not only in generating sequences but in navigating the geometry of a learned representational space: generation is a consequence of navigation, and their apparent creativity emerges from movement through learned manifolds of meaning.

In the molecular domain, an analogous revolution has unfolded, driven by a succession of generative architectures of increasing sophistication. Variational autoencoders applied to SMILES strings enabled the first continuous latent-space representations of molecular structure, in which interpolation between two molecular embeddings yielded chemically plausible intermediate structures.^3^ Graph neural network-based generative models extended this approach to the graph-structured representation of molecules, enabling the generation of novel molecular graphs with controlled properties without the information loss inherent in SMILES linearization.^28^ Normalizing flow models—including GraphAF and the MoFlow architecture on which our group’s TumFlow model is based—offered invertible mappings between molecular graph space and a simple prior distribution, enabling both efficient sampling and exact likelihood computation.^29^

Diffusion models, most recently, have demonstrated exceptional capacity for molecular generation in three-dimensional space. Models such as the equivariant diffusion model of Hoogeboom and colleagues generate molecules by learning to reverse a diffusion process that progressively adds noise to three-dimensional molecular conformations, producing structures that are not only chemically valid and structurally diverse but also consistent with physically plausible geometric constraints.^25^ These models learn the organization of three-dimensional chemical space from large databases of known molecules and generate new candidates by navigating that learned geometry through iterative denoising—a process that bears a striking formal resemblance to the alchemical pathways of Free Energy Perturbation, as we discuss in the next section.

Yet the analogy has scientifically important limits. A language model’s output is judged by human readers; a molecular model’s output must survive synthesis, thermodynamics, pharmacokinetics, and biological assay—constraints far more stringent than grammaticality. A sentence may be meaningful without being true; a molecule cannot be useful without obeying the laws of chemistry and biology.

### 4.1 TumFlow: A Case Study in Navigating Chemical Meaning

These principles are illustrated by a growing body of generative approaches from many groups, spanning variational autoencoders, reinforcement learning, and diffusion models.^3,28–30^ As one concrete example, drawn from our own work so that we can discuss its internals candidly, we consider TumFlow, a normalizing-flow model for anticancer molecule generation.^31^ Rather than recounting its architecture—available in the primary publication—we focus on what it reveals about the navigational logic at the heart of this Perspective.

TumFlow navigates a learned latent representation of molecular space by following the gradient of a predicted biological-activity score, moving from a known active toward regions of higher predicted efficacy. This is navigation through a manifold of chemical meaning—the molecular analogue of a language model traversing semantic space—generation as directed movement rather than random sampling.

The outcomes were revealing in equal measure of promise and limitation. TumFlow generated novel structures absent from public databases, and in one case converged independently on a compound previously validated for anticancer activity—strong evidence that learned chemical geometry can encode genuine biological relevance. In its initial formulation, however, the model also displayed a tendency to propose structures of limited synthetic accessibility—a well-known limitation of generative approaches that do not explicitly incorporate synthesizability, and one that can be substantially mitigated by coupling generation to synthetic-accessibility scoring^32^ or retrosynthetic planning.^6–8^ This tension—between navigating the statistical geometry of known chemical space and the functional geometry of pharmacologically tractable space—remains an active and central challenge of molecular AI, and TumFlow makes it visible with unusual clarity.

### 4.2 Alchemical Transformations and Latent Interpolations

A striking conceptual bridge between classical simulation and generative modeling is Free Energy Perturbation (FEP). In FEP, two molecular states are connected by a continuous alchemical pathway parametrized by a coupling variable λ.^33^ As λ runs from 0 to 1, the system traverses intermediate states that need not correspond to any real molecule, yet define a thermodynamically meaningful trajectory through molecular space.

FEP can thus be seen as an early framework for reasoning about continuous molecular transformations—long before molecular embeddings or generative AI.^34^ The λ parameter can be viewed as an early conceptual analogue of the latent coordinate of a generative model: a continuous variable parametrizing a trajectory through a structured space.

Latent-space interpolation has the same structure. Interpolating between two molecular embeddings traces a path through a learned manifold along which properties vary smoothly, even when intermediate structures are not individually synthesizable.^3^ Both frameworks treat molecular transformation as continuous navigation rather than discrete jumps (Figure 4).^3^

**Figure 4:**
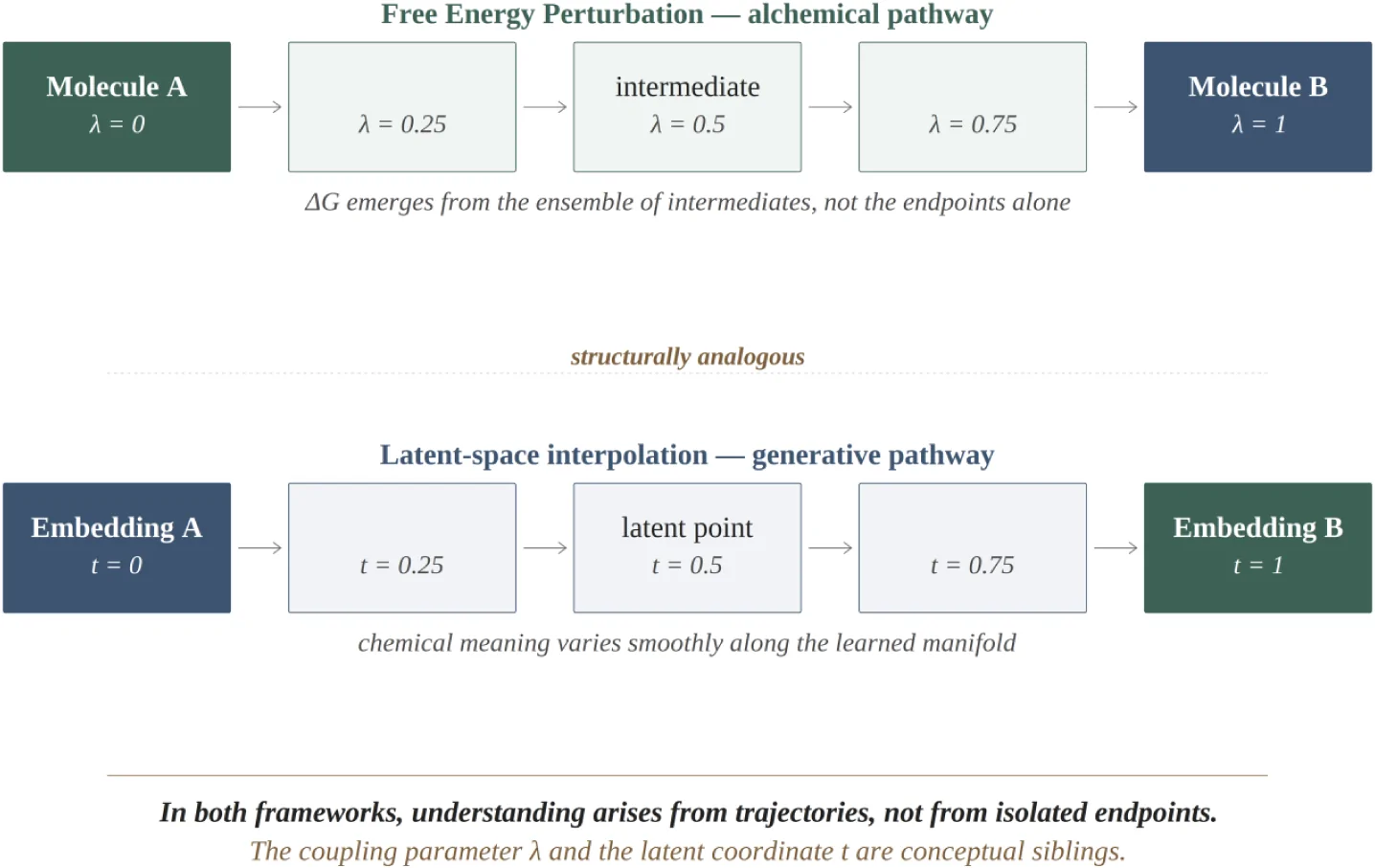
Conceptual parallel between Free Energy Perturbation (FEP) and latent-space interpolation. In both, two molecular states are connected by a continuous pathway whose intermediate points carry the scientific meaning; the alchemical coupling λ and the latent coordinate t are conceptual siblings.

The value in both cases lies in the pathway, not the end points. Recent work connecting physically grounded representations to learned latent spaces suggests that FEP and generative AI are not competing traditions but complementary strategies for exploring chemical meaning—one grounded in thermodynamics, the other in learned geometry—whose convergence may guide generation along physically meaningful trajectories.^35^

## 5 Human Intuition in the Loop—What Machines Cannot (Yet) Replace

This history might suggest that the formalization of chemical knowledge has rendered human intuition superfluous. This conclusion would be mistaken, and understanding why is essential for the responsible deployment of AI in chemical research.

Human chemical intuition is not merely pattern recognition over known molecules. It appears to draw substantially on causal reasoning embedded in an understanding of physical principles, synthetic constraints, biological mechanisms, and experimental practice. An experienced medicinal chemist does not merely estimate a structure’s likelihood under a learned distribution; she asks whether it can be synthesized affordably, whether its metabolic liabilities are manageable, whether its mechanism is plausible given the target biology, and whether its novelty supports a patent position—judgments drawing on knowledge across many domains, only partially captured in training data.^36^

A model trained on existing compounds will faithfully reproduce the statistical regularities of known drug-like molecules; and without appropriate guidance it may struggle to avoid the synthetic cul-desacs, metabolic liabilities, and off-target interactions that experienced chemists navigate by intuition and principle. Recent approaches that couple generative models with retrosynthetic analysis, property predictors, and multiparameter optimization have begun to mitigate these limitations, though integrating the full range of such considerations within a single design process remains challenging.

Scientific creativity—formulating genuinely new hypotheses, recognizing unexpected analogies, conceiving architectures with no precedent—is not well described as interpolation within a learned geometry. The great discoveries expanded what space was conceivable rather than traversing a known one. Current generative models are powerful navigators of known chemical space; the extent to which they can also function as explorers of genuinely new territory—rather than sophisticated interpolators of it—remains an open and actively debated question.^37^

The discovery of halicin offers the clearest illustration of what human–machine collaboration can achieve, and where its boundary lies.^37^ A deep-learning model proposed halicin because its features correlated statistically with antibacterial activity—an approach unlikely to have been prioritized through conventional medicinal-chemistry heuristics, given that its structural class had no antibiotic precedent. But it was human researchers who recognized the proposal’s significance, tested it, and interpreted its unprecedented mechanism. AI proposes, humans interpret, discovery emerges—not a transitional arrangement but the appropriate architecture for a domain whose success criteria are set by biological reality.

Operationalizing this architecture at scale requires the right infrastructure. MolVE, an open-source platform developed in our group, supports distributed, asynchronous expert evaluation of AI-generated molecules, presenting machine-learning predictions alongside interactive visualizations and structured qualitative feedback.^38^ By surfacing cases where human judgment and machine scoring diverge, and exporting expert assessments as machine-readable training data, MolVE provides the infrastructure for a closed feedback loop—the operational infrastructure for the chemist-in-the-loop paradigm. The paradigm and its implementation are shown in Figure 5. Comparable efforts elsewhere—for example, active-learning and human-in-the-loop frameworks that couple expert feedback to generative models^39^—point in the same direction, underscoring that this is a broad and collective research program rather than a single-group endeavor.

**Figure 5:**
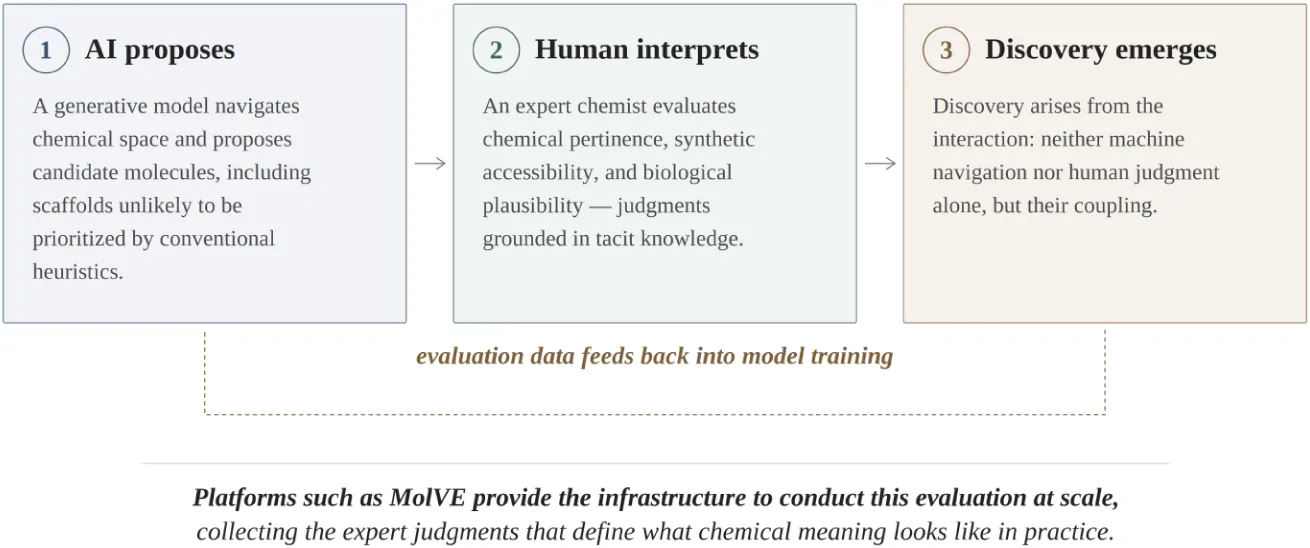
Chemist-in-the-loop paradigm and its operational implementation. AI proposes candidates, the expert interprets and evaluates them, and discovery emerges from their interaction; MolVE provides the infrastructure to conduct this at scale.

Our own experience with TumFlow illustrates these limitations concretely: the model navigated chemical space efficiently and proposed novel candidates, but human expertise remained essential at every stage—filtering for synthetic accessibility, assessing druglikeness,^40^ and interpreting predicted activity against known biology. The machine expanded the space of candidates; the judgment of which were worth pursuing remained irreducibly human.

The appropriate framing is therefore not the autonomous system that replaces the chemist, but the augmented chemist who explores a far larger region of chemical space while bringing causal reasoning and physical intuition to the evaluation of the machine’s proposals. The chemist-in-the-loop is not a concession to current limitations; it is, at least for now, a well-suited epistemic architecture for a domain where tacit knowledge, causal reasoning, and experimental feedback have so far proven difficult to reduce to statistical pattern-matching alone. How this division of labor will evolve as AI systems become more capable remains an open question.

## 6 Outlook—Toward a Unified Theory of Chemical Language

### 6.1 Emerging Directions in Molecular AI

The convergence of chemical representation, molecular simulation, and machine learning points toward what might be called a unified theory of chemical language.

Lavoisier provided chemistry a vocabulary; Kekulé, a grammar; Weininger, a writing system. Molecular AI is now learning to speak that language, with diffusion models acting as navigation engines over learned manifolds of chemical meaning.

Several directions seem especially promising. First, molecular foundation models—pretrained on diverse chemical data and fine-tuned per task—mirror the foundation-model paradigm in NLP and promise rapid adaptation with limited labeled data.^31,41^

Second, integrating chemical language models with structured domain knowledge—reaction databases, protein structures, bioassay results—offers a path toward models whose predictions are more tightly constrained by physical and mechanistic knowledge rather than by statistical regularities alone.^42^ Systems such as AlphaFold, which combine learned representations with structural and evolutionary constraints, illustrate how far physically grounded integration can extend the reach of such models.

Third, aligning learned chemical geometry with functional topology remains a central open problem: models that organize chemical space by mechanism and activity, rather than by structural similarity alone, would represent a qualitatively more powerful chemical intelligence. The framework of chemical meaning makes this goal precise—learning representations in which geometric proximity tracks similarity in chemical meaning.

Fourth, interpretable human-AI interfaces will determine whether these gains are realized in practice. Platforms such as MolVE show this is already accessible, translating chemical-space geometry into the vocabulary of chemical intuition and enabling the chemist-in-the-loop paradigm at scale.^38^

Fifth, and most speculatively, AI may itself participate in designing new chemical languages—representation systems optimized not for human convenience but for functional geometric fidelity to chemical meaning. Current notations were designed to encode structure unambiguously, not to make chemical meaning geometrically accessible to learning algorithms; a representation minimizing the distance between functionally similar molecules could yield geometries far more faithful to chemical meaning.

Just as chemistry progressed from Lavoisier’s notation to Kekulé’s structural formulas, from SMILES to SELFIES—the next chapter may involve representation systems that are not designed by human intuition alone but codeveloped with AI, iteratively refined against the criterion of functional geometric fidelity. In this vision, the chemical language of the future would not be chosen from among existing alternatives, but discovered—through the same generative and optimization processes that molecular AI already applies to molecular structures—as the representation that most faithfully maps chemical meaning into navigable geometric space. Language and chemistry, which have run in parallel throughout this Perspective, would then converge not only in their computational treatment, but in their very method of symbolic innovation.

Ultimately, progress in molecular AI may be evaluated by increasingly demanding versions of the Chemical Imitation Game, in which machine-generated proposals are assessed not for validity alone, but for their capacity to exhibit the forms of chemical reasoning traditionally associated with expert practitioners—the scaffold intuition, the synthetic judgment, the biological plausibility, and the creative originality that define chemical expertise. Each incremental closing of that gap will mark a genuine advance; the distance that remains will define the next generation of problems.

### 6.2 The Story of Representation

It is worth crystallizing the central proposition in its sharpest form. Chemical meaning is to molecules what semantic meaning is to words: an emergent property arising from relationships, context, and function rather than from isolated symbols. A word has no meaning outside the language system; a molecule has no chemical meaning outside the physical and biological context that determines its behavior. Both are relational constructs that can, in principle, be approximated geometrically—the conceptual core of the unified theory of chemical language.

At its deepest level, this is not a story about machines but about representation. Every advance described here—from Dante’s vernacular to Lavoisier’s nomenclature, from Kekulé’s formulas to molecular embeddings—expanded the capacity to describe, communicate, and explore increasingly complex spaces of possibility.

Artificial intelligence is the latest chapter in this progression—not a replacement for human understanding but an extension of its reach into regions of chemical space too vast for unaided cognition. Chemical meaning is not a fixed property waiting to be discovered, but a construct emerging from the interaction of molecular structure, physical context, and human interpretation.

From Dante and Lavoisier to large language models and molecular diffusion models, the trajectory is one of progressive formalization that has consistently expanded human capability without replacing human judgment. The most productive framing for molecular AI is therefore not how to automate discovery, but how to design representations and tools that most effectively amplify the distinctive contributions of human expertise—intuition, causality, and creativity—in exploring the vast landscape of molecular possibility.

## 7 Closing Statement

From words to molecules, the story is one of progressive formalization: symbols become representations, representations become geometries, and geometries become navigable spaces. Yet the ultimate purpose of this progression has never been the replacement of human judgment. It has been the expansion of human possibility.

The Chemical Imitation Game remains open. Current molecular AI navigates chemical space with remarkable efficiency, yet whether expert chemists can consistently distinguish machine-generated proposals from human-designed candidates remains an open empirical question. That gap is not a failure; it is a precise definition of the frontier. Closing it will require not only better models, but better chemical languages, better geometric representations of chemical meaning, and a deeper understanding of what it means for a molecule to be not merely valid, but meaningful. If the Chemical Imitation Game is to be taken seriously as an evaluative framework for progress in molecular AI, platforms such as MolVE provide the operational infrastructure to conduct it at scale—systematically collecting the expert judgments that define what chemical meaning looks like in practice, and feeding them back into the machines that are learning to navigate it.

Artificial intelligence does not replace chemical intuition; it extends humanity’s capacity to explore the vast landscape of molecular possibility. In doing so, it continues a tradition that began long before computers—from Dante’s vernacular to Lavoisier’s nomenclature, from Kekulé’s structural insight into Weininger’s SMILES—rooted in a simple but transformative idea: that by finding better ways to represent the world, we may also discover better ways to understand it. Chemical meaning—like linguistic meaning before it—is not discovered by machines alone. It is constructed, in the space between molecular structure and human interpretation, by the same collaborative intelligence that has always driven science forward. The game continues.
